# Early onset of inflammation during ontogeny of bipolar disorder: the *NLRP2* inflammasome gene distinctly differentiates between patients and healthy controls in the transition between iPS cell and neural stem cell stages

**DOI:** 10.1038/tp.2016.284

**Published:** 2017-01-24

**Authors:** D Vizlin-Hodzic, Q Zhai, S Illes, K Södersten, K Truvé, T Z Parris, P K Sobhan, S Salmela, S T Kosalai, C Kanduri, J Strandberg, H Seth, T O Bontell, E Hanse, H Ågren, K Funa

**Affiliations:** 1 Sahlgrenska Cancer Center, Institute of Biomedicine, Sahlgrenska Academy, University of Gothenburg, Gothenburg, Sweden; 2 Institute of Neuroscience and Physiology, Department of Physiology, Sahlgrenska Academy, University of Gothenburg, Gothenburg, Sweden; 3 Oncology Laboratory, Department of Pathology, Sahlgrenska University Hospital, Gothenburg, Sweden; 4 Institute of Neuroscience and Physiology, Section of Psychiatry and Neurochemistry, Sahlgrenska Academy, University of Gothenburg, Gothenburg, Sweden; 5 Bioinformatics Core Facility, Sahlgrenska Academy, University of Gothenburg, Gothenburg, Sweden; 6 Institute of Biomedicine, Department of Medical Genetics, Sahlgrenska Academy, University of Gothenburg, Gothenburg, Sweden; 7 Department of Clinical Pathology and Cytology, Sahlgrenska University Hospital, Gothenburg, Sweden

## Abstract

Neuro-inflammation and neuronal communication are considered as mis-regulated processes in the aetiology and pathology of bipolar disorder (BD). Which and when specific signal pathways become abnormal during the ontogeny of bipolar disorder patients is unknown. To address this question, we applied induced pluripotent stem cell (iPSC) technology followed by cortical neural differentiation on adipocyte-derived cells from BD type I patients (with psychotic episodes in psychiatric history) and healthy volunteers (controls). RNA sequencing in iPSC and cortical neural stem cell (NSC) lines were used to examine alterations between the transcriptomes from BD I and control samples during transition from the pluripotent stage towards the neural developmental stage. At the iPSC stage, the most highly significant differentially expressed gene (DEG) was the *NLRP2* inflammasome (*P*=2.66 × 10^−10^). Also among 42 DEGs at the NSC stage, *NLRP2* showed the strongest statistical significance (*P*=3.07 × 10^−19^). In addition, we have also identified several cytoskeleton-associated genes as DEGs from the NSC stage, such as *TMP2*, *TAGLN* and *ACTA2;* the former two genes are recognised for the first time to be associated with BD. Our results also suggest that iPSC-derived BD-cortical NSCs carry several abnormalities in dopamine and GABA receptor canonical pathways, underlining that our *in vitro* BD model reflects pathology in the central nervous system. This would indicate that mis-regulated gene expression of inflammatory, neurotransmitter and cytoskeletal signalling occurs during early fetal brain development of BD I patients.

## Introduction

Bipolar disorder (BD) is a severe and chronic disorder characterised by the cyclic occurrence of episodes of mania and depression. BD is also associated with significant disability, morbidity, and cognitive impairment.^1, 2^ It is frequently comorbid with several medical conditions including cardiovascular and metabolic diseases.^2, 3^ However, the connections between extra- and intra-cerebral pathologies are largely unknown. Nevertheless, the co-occurrence of autoimmune diseases has been reported, that is, systemic lupus erythematosus,^4^ multiple sclerosis^5, 6^ and autoimmune thyroiditis,^7^ as well as altered levels of circulating inflammatory cytokines, including interleukin (IL)-6, TNF-α, IFN-γ and IL1-β.^8^ A review of immunological factors in the pathophysiology of BD describes a major imbalance in inflammatory cytokines.^9^ These findings suggest the presence of immunological activation in BD of adult individuals. However, when chronic inflammation starts during the lifespan of BD patients is unknown.

The molecular and cellular mechanisms contributing to BD initiation and progression are poorly understood. The heritability of BD is estimated to be as high as 90%, suggesting a strong genetic basis.^10^ In light of this high heritability, genome-wide association studies have been used to identify genetic pathways associated with BD.^11^ In fact, several abnormally regulated genes have been identified in adult BD patient samples.^12^ It is not known when a BD-associated mis-regulated gene expression would start during the lifespan of BD patients.

Combining iPSC technology^13, 14, 15^ and neural differentiation^16^ of cells from patients and healthy controls allows *in vitro* modelling of human neurodegenerative disorders,^17, 18, 19, 20, 21, 22, 23^ as well as complex genetic neuropsychiatric disorders.^24, 25, 26, 27^ However, the polygenic nature of BD and the pronounced genetic overlap between schizophrenia and BD type I (BD I, with manic episodes in psychiatric history)^11, 28, 29^ requires further investigations to find out when and which genes as well as which signalling pathways are abnormally regulated during neural development in BD. Thus, there is a need to investigate the global transcriptome of BD I patients and healthy controls during the early stages of neural development, that is, the neural stem cell (NSC) stage.

For this purpose, we used disease modelling using iPSC technology and cortical neural differentiation of adipocytes obtained from euthymic BD I patients under medical treatment as well as from healthy individuals. Subsequently, we performed RNA sequencing (Seq), providing high sensitivity with the capacity to detect low-copy transcripts. We established a human BD model system to understand when and how the BD I-associated genes manifest, by comparing RNAs at iPSC and NSC stages of six BD I patients and four healthy controls (including one from Cellartis DEF-hiPSC line). We found several differentially expressed genes (DEGs) involved in immune responses at the NSC stage. Remarkably, one of these genes, *NLRP2*, was identified as a DEG as early as in iPSC stage when compared with the controls. Furthermore, the DEGs were uploaded to a signalling pathway analysis, demonstrating dopamine and GABA receptor canonical pathways to be abnormally expressed already in the NSC stage. Our results not only provide further evidence that BD I is a developmental disorder with polygenetic disposition, but also reveal that mis-regulated expression of genes involved in inflammation and neurotransmitter systems occur already during the early stages of fetal neural development in BD I patient cells.

## Materials and methods

### Generation of hiPSC lines and culture conditions

Abdominal subcutaneous adipose tissue was isolated and primary adipocyte cell lines were established.^30^ Starting with adipocytes from patients (B1, B2, B3, B4, B5, B6) and controls (C1, C2 and C3), iPSCs were generated and characterised by Cellectis (formerly Cellartis, presently Takara Clontech, Shiga, Japan). As an additional and study-independent control, one Cellartis DEF-hiPSC ChiPSC4 line (C4) was used. All lines were cultured under feeder-free conditions in Cellartis DEF-CS (Takara Bio Europe, Gothenburg, Sweden) at 37 °C in a humidified atmosphere of 5% CO_2_ in air. Primary adipocytes were cultured as described earlier^30^ without supplement of insulin, dexamethasone or other reagents that maintain adipocyte characteristics.

To create an *in vitro* BD model system, adipocyte cell lines were thus originally derived from abdominal subcutaneous fat samples of altogether 35 BD (11 BD I, 7 BD II and 17 BD non ultra descriptus) patients and 38 healthy controls, as earlier described.^30^ From these cell lines, six BD I and three healthy controls were selected on technical grounds. The six BD I patients (three females, three males) donating abdominal adipocytes were all euthymic at the time of examination with GAF scores (±s.d.) 69.2±13.6, Young Mania Rating Scale 0.7±1.6 and Montgomery–Åsberg Depression Rating Scale 5.7±5.1. They all had suffered from at least one psychotic episode in their psychiatric history. All have Caucasian origins. Their age at first mood episode compared with their current age was 12/53, 18/39, 17/44, 15/47, 15/47 and 21/32 years. Four were currently medically mood-stabilised with lithium, four with antipsychotics and two with antiepileptics. Three controls were healthy females, age 27, 26 and 29 years. One ChiPSC4 line (C4) male control was added.

### Directed differentiation of hiPSC

The hiPSCs lines were plated on COAT1 (Takara Bio Europe) coated 12-well plates in feeder-free conditioned Cellartis DEF-CS medium (Takara Bio Europe) until confluence. Neural induction was initiated by changing the culture medium to 1:1 mixture of N2 media, consisting of DMEM/F12 GlutaMAX (Life Technologies, Carlsbad, CA, USA), N2 supplement (Life Technologies), 5 μg ml^−1^ insulin (Sigma-Aldrich, St. Louis, MO, USA), 1 mm Ultra glutamine (Lonza, Basel, Switzerland), 100 μm non-essential amino acids (Gibco, Carlsbad, CA, USA), 100 μm 2-mercaptoethanol (Gibco), 50 U ml^−1^ penicillin and streptomycin (Lonza), and B27 media (Neurobasal; Life Technologies), consisting of B27 with vitamin A (Life Technologies), 2 mm Ultra glutamine (Lonza), 50 U ml^−1^ penicillin and streptomycin (Lonza) supplemented with 1 μm Dorsomorphin (Tocris Bioscience, Bristol, UK) and 10 μm SB431542 (Tocris Bioscience). Neural induction media was replaced every day for 8–10 days until a uniform neuro-epithelial sheet was observed, and then both iPSC and neuro-epithelial cells were frozen in RNA protect (Qiagen, Redwood City, CA, USA) for miRNA and transcriptome analysis. Neuro-epithelial cells were collected by dissociation with Dispase (Life Technologies) and aggregates were re-plated on laminin-coated plates and maintained in neural maintenance media. On appearance of rosette structures, NSCs were expanded by supplementing media with 20 ng ml^−1^ FGF2. After a further 4 days, FGF was withdrawn and the cultures were passaged using Accutase and maintained in a neural maintenance media until frozen at day 23–30 post initiation of neural induction. For neurogenesis, NSCs were cultured on poly-l-ornithine/laminin-coated dishes on a feeder layer of human astrocytes. Neural maintenance media was changed every second day.

### Immunostaining

For immunofluorescence, iPSCs, neuro-epithelial cells and NSCs were collected by dissociation with Tryple Select (Life Technologies), Dispase (Life Technologies) and Accutase, respectively. After counting, the 5.0 × 10^6^ iPSC were re-plated onto eight-well chamber slides coated with laminin 521 (BioLamina, Sundbyberg, Sweden), and cultured overnight in Cellartis DEF-CS (Takara Bio Europe). Neuro-epithelial aggregates were re-plated on laminin-coated coverslips and maintained in neural maintenance medium until appearance of rosette structures. NSCs were re-plated onto eight-well chamber slides coated with laminin and maintained in neural maintenance medium for 60 days. The cells were fixated with 4.0% paraformaldehyde/phosphate-buffered saline (PBS) for 20 min, washed in 0.1% Tween 20/PBS, and permeabilised with 0.25% Triton X-100/PBS for 10 min and blocked by 0.1% Tween 20/0.3 m glycine/10% normal donkey or goat serum/PBS for 1 h. The primary antibodies used were anti-Oct-3/4 (H-134) (1:200, sc-9081; Santa Cruz Biotechnology, Dallas, TX, USA), anti-SSEA4 (MC813) (1:50, ab16287, Abcam, Cambridge, UK), anti-sialyl-lactotetra (TR4) (1:500), anti-Pax6 (1:200, ab2237; Millipore, Solna, Sweden), anti-Otx2 (1:200, ab9566; Millipore), anti-Nestin (ab22035; Abcam), anti-Ki67 (1:200, sc-23900; Santa Cruz). For secondary antibodies, Alexa Fluor 488 conjugated donkey anti mouse IgG and Alexa Fluor 555 conjugated donkey anti rabbit IgG (1:1000, Life Technologies) were used, which were diluted in blocking solution and added for 2 and 1 h, respectively, each followed by washes in 0.1% Tween 20/PBS. The nuclei were counterstained with Hoechst. The slides were mounted and analysed on Nikon Eclipse Ni-E automated upright microscope (Tokyo, Japan) equipped with Orca Flash 4.0 camera (Hamamatsu Photonics, Shizuoka, Japan), using Plan Apo lambda × 20/0.75 or Plan Apo lambda 60 (Nikon) objectives.

### Electrophysiology recordings

For electrophysiological experiments, NSC were plated on laminin-coated Ibidi μ-dishes (Ibidi, Munich, Germany) and maintained in a neural maintenance media for 30 to 127 days. The μ-dishes were mounted under a microscope (Nikon E600FN) where the cells were perfused (2–3 ml min^−1^) with artificial cerebrospinal fluid containing 1 mm NaH_2_PO_4_, 123 mm NaCl, 26 mm NaHCO_3_, 3 mm KCl, 2 mm MgCl_2_, 1 mm CaCl_2_ and 10 mm d-glucose. The artificial cerebrospinal fluid was continuously bubbled with gas containing 95% O_2_ and 5% CO_2_. Patch-clamp recordings were performed on cells visually identified using infrared differential interference contrast video microscopy. The data were acquired with a patch-clamp amplifier (EPC-10; Heka Elektronik, Lambrecht, Germany) at a sampling frequency of 20 kHz and filtered at 2.9 kHz. Patch pipettes (3–7 MΩ) were pulled using a horizontal Flaming/Brown micropipette puller (P-97; Sutter Instrument Company, Novato, CA, USA). The pipette solution contained 127 mm K-gluconate, 8 mm KCl, 10 mm Hepes, 15 mm phosphocreatine, 4 mm Mg-ATP and 0.3 mm Na-GTP (pH 7.2, 295 mOsm). Action potentials were recorded in current clamp at –70 mV and a series of 15 hyperpolarising and depolarising current injections (–20 to 50 pA and –20 to 120 pA, 300 ms duration) were applied. Analyses of action potentials were done using Igor Pro (WaveMetrics, Lake Oswego, OR, USA). To record spontaneous excitatory postsynaptic currents and inhibitory postsynaptic currents, the cells were voltage clamped at –70 and 0 mV, respectively. Analyses of spontaneous excitatory postsynaptic currents/inhibitory postsynaptic currents were performed using the Mini Analysis Program (Synaptosoft, Fort Lee, NJ, USA). Series resistance was monitored using a 10 mV hyperpolarising pulse and was not allowed to exceed 30 MΩ.

### RNA extraction

RNA was isolated from iPSC and NSC using total RNA purification plus kit (Norgen, Thorold, ON, Canada). To ensure that the samples were not contaminated with genomic DNA, an additional DNase I digestion step was performed with RNase-free DNase (Norgen) according to the manufacturer’s instructions. RNA quantity and quality were determined using an ND-1000 spectrophotometer and Agilent RNA 6000 Nano Kit (Santa Clara, CA, USA), respectively. RNA integrity number values above 8.0 were accepted for further analysis. The RNA samples were stored at –80 °C.

### Reverse transcription and real-time PCR

Complementary DNA synthesis was performed with iScript Reverse Transcription Supermix for reverse transcription and quantitative PCR (RT-qPCR; Bio-Rad). Endogenous messenger RNA (mRNA) levels were measured by RT-qPCR analysis using SsoAdvanced Universal SYBR Green Supermix (Bio-Rad) according to the manufacturer’s instructions. Each sample was analysed in triplicate using the following oligonucleotide pairs: *RPLP* F: 5′-CGACAATGGCAGCATCTACAAC-3′ and R: 5′-CGGACACCCTCCAGGAAG-3′ *GUSB* F: 5′-AATCACTATGCGCATCAACAACA-3′ and R: 5′-TTGGGATACTTGGAGGTGTCA-3′ and *NLRP2* Hsa Prime PCR assay (Bio-Rad). All the primer pairs yielded a single product as confirmed by dissociation curve analyses, and gave no product in the no-template control. The data analysis was performed in GenEx 6.1 (TATAA Biocenter, Gothenburg, Sweden), using a preset calculation, subtraction of gDNA background with Validprime. The data were then exported to Excel and normalised with the ΔΔCt method, results presented as fold change.

### Transcriptome sequencing and data analysis

Isolation of mRNA, library preparation and transcriptome sequencing using Illumina HiSeq2000 were performed by BGI Tech Solutions (Hong Kong). The data files made in BGI were analysed in-house as follows. Before mapping of the genome, adaptors were cut and the raw reads were subjected to quality control, trimming and filtering. The Trim Galore (0.3.3) tool, a wrapper tool around Cutadapt^31^ and FastQC, was used for this purpose with default settings (phred score cut-off=20). Trimmed reads were mapped to the human (hg19) reference genome using STAR (2.4.0f).^32^ HTSeq (0.5.3p3; ref. 33) was used to quantify number of reads/gene using a list of annotated genes downloaded from Ensembl (version 75).^34^ The DESeq2 package^35^ from Bioconductor (Cambridge, MA, USA) was used to determine DEGs between samples derived from BD patients and healthy controls. Furthermore, analysis of DEGs was performed using DESeq2 between iPSC and NSC stages for BD and control cell lines, respectively.

### Pathways and network analysis

Ingenuity pathway analysis software (Qiagen) was used to assess the disease-associated DEGs for the enrichment of gene networks, canonical pathways and biological processes relevant to the pathogenesis of BD. A list of the DEGs between BD and controls were uploaded to ingenuity pathway analysis, and analysis was performed at significance level of *P*<0.05 and log2-fold enrichment >1.8.

## Results

### iPSC generated from adipocyte cell lines from six BD I patients and four healthy controls

**Figure 1:**
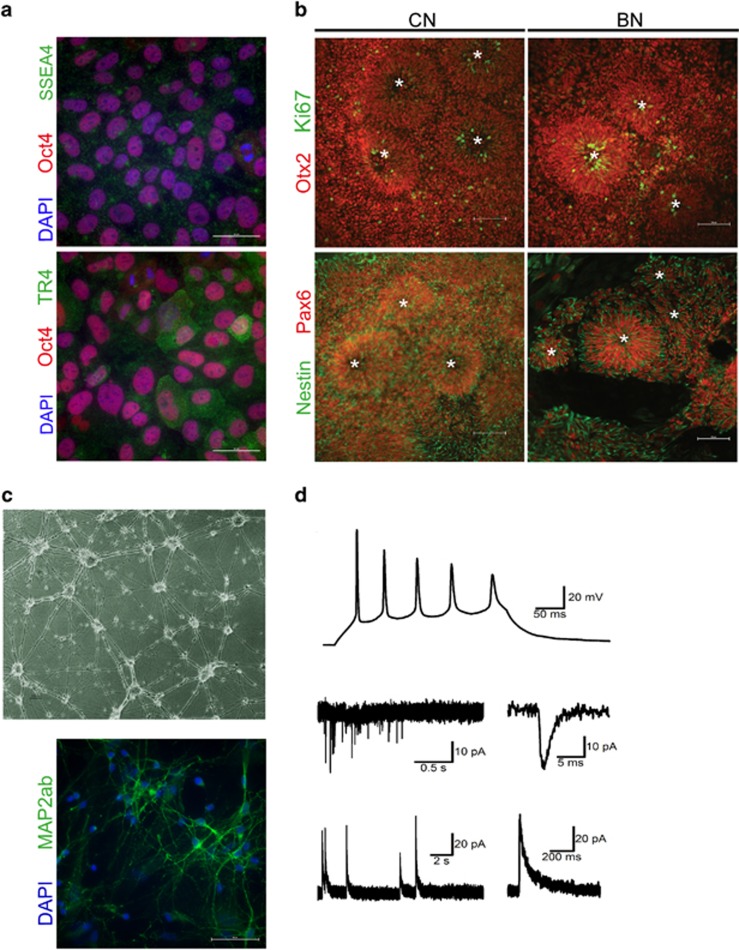
Directed differentiation of human iPSCs, into cortical stem and progenitor cells followed by cortical neurogenesis. (**a**) Representative images of iPSCs confirming presence of pluripotency markers Oct4 (red), SSEA4 (upper, green) and syalys-lactotetra (TR4) (lower, green). Nuclei were counterstained with Hoechst. Scale bars represent 50 μm. (**b**) Representative images of NSC forming rosettes derived from healthy control (CN) and bipolar patient (BN) specific iPSCs. NSCs exhibit a rosette-like growth pattern (indicated with a star in rosetta lumen) of Ki67 positive (upper, green) proliferating cells and stain positive for Otx2 (upper, red) and cortical stem cell marker Pax6 (lower, red) as well as Nestin (lower, green). Scale bars represent 100 μm. (**c**) NSCs can be differentiated into neurons expressing MAP2-AB (green). Nuclei were counterstained with Hoechst. Scale bars represent 50 μm. (**d**) Whole-cell current-clamp and voltage-clamp recordings were performed at different time points on NSCs differentiated into cortical-like neurons. (Upper) Example sweep showing evoked action potentials in response to a depolarising current pulse (300 ms). (Middle) Example sweep of voltage-clamp recordings showing spontaneous EPSCs recorded at –70 mV. (Lower) Example sweep of voltage-clamp recordings showing spontaneous IPSCs recorded at 0 mV. EPSC, excitatory postsynaptic current; iPSC, induced pluripotent stem cell; IPSC, inhibitory postsynaptic current; NSC, neural stem cell.

Nine of the established adipocyte cell lines mentioned above (the six BD I patients and four healthy controls) were selected for reprogramming into iPSC lines^36^ using a non-integral episomal reprogramming technology to avoid gene alteration.^37, 38^ Briefly, iPSC colonies were initially selected by morphology, passaged several times and expanded before characterisation. Pluripotency of all lines was confirmed by the presence of the pluripotency markers, that is, TRA-1-60, TRA-1-81, SSEA4, OCT4 and SOX2 (data not shown). As a further test of pluripotency, undirected differentiation of all nine iPSC lines into embryoid bodies and subsequent immunofluorescence analysis using β-tubulin, HNF4α and ASMA confirmed the presence of the cells from all three germ layers, that is, ectoderm, endoderm and mesoderm (data not shown). After expansion in our laboratory, presence of pluripotency markers Oct4, SSEA4 and sialy-lactotetra (TR4)^39^ was confirmed by immunofluorescence (Figure 1a). These results demonstrate the successful generation of pluripotent iPSC lines from BD patients and healthy controls.

### Characterisation of control and BD NSCs

To specifically direct differentiation of iPSC lines into cortical stem and progenitor cells (or neural lineage), all nine iPSC lines and the study-independent control line, ChiPSC4, were plated as single cells and subsequently neuralised by dual inhibition of SMAD signalling in combination with retinoid signalling.^16^ Eight to 10 days post neural induction, confirmation of directed iPSC differentiation was performed by expression of *TERT* as well as the transcription factors *OCT4*, *NANOG*, *FOXG1* and *PAX6*.

Using this approach, decreased expression of pluripotency markers *JARID1*, *NANOG* and *TERT* was confirmed, while expression of *PAX6* and *FOXG1* was increased, indicating successful neural induction in all 10 iPSC lines (Table 1). In addition to the expression of unique cortical stem and progenitor cells transcription factors, all 10 lines were capable of forming neural rosette structures with a morphology characteristic of early neuro-epithelium and a feature of ES/iPSC-derived NSC.^40, 41^ Immunofluorescence analysis of these rosette structures confirmed their cortical identity by high levels of *PAX6* and *OTX2* as well as *NESTIN* (Figure 1b). In addition, there was no detectable difference in the labelling of either control or BD NSC by the cell cycle marker Ki67 (Figure 1b). These results demonstrate the successful differentiation of iPSCs into NSCs.

### NSCs have the capacity to make functional neurons

To further investigate the *in vitro* BD model system, we asked whether generated NSCs are capable of differentiating into functional neurons. Thus, we differentiated the NSCs into cortical-like neurons expressing MAP-AB (Figure 1c), using an established protocol.^16^ Whole-cell current-clamp and voltage-clamp recordings were then performed at different time points between 51 and 147 days after start of neural induction. In 49 of 58 investigated cells (84%), action potential firing could be induced using a depolarising current injection (300 ms) (Figure 1d). In 16 of the 58 cells (28%), spontaneous synaptic events could also be detected. All the spontaneous synaptic events were seen in cells showing action potentials (16 of 49 cells, 33%).

Both excitatory postsynaptic currents and inhibitory postsynaptic currents could be detected in five cells, only excitatory postsynaptic currents in seven cells and only iPSCs in four cells (Figure 1d). Taken together, these data indicate that the generated NSCs can be further differentiated into functional neurons of both excitatory and inhibitory type and further strengthen the capacity of the generated *in vitro* BD model system.

### Comparison of the transcriptome of BD and control cells at iPSC and NSC stages

Having established the capacity of the system, we addressed developmental differences between BD and control cell lines at the transcriptome level. To this end, RNA was isolated from both iPSC (BI, CI) and NSC (BN, CN) stages for the six BD I and four control lines and subjected to RNA-Seq analysis. All the samples from individual stages, that is, iPSC and NSC, clustered together, revealing clear similarity within iPSC and NSC stages regardless of origin (Supplementary Figure). These results indicate that samples from each stage are very closely related and that each stage is likely to exhibit functionally distinct and unique transcriptomes.

A total of 3302 and 3187 transcripts from healthy controls and BD I patients, respectively, were differentially expressed at log2-fold enrichment 2 or higher, with *P*-values in the control and BD cell lines between different stages (Supplementary Information 1 and 2). With neural induction, expression of genes associated with pluripotency, such as *JARID1*, *NANOG* and *TERT,* was downregulated, while expression of genes associated with neuronal patterning was increased to a similar extent in BD as well as in control cell lines (Table 1).

These data suggest that with neural induction, cells express different sets of signalling molecules compared with iPSCs. In addition, no significant difference of expression of genes involved in neuronal patterning or NSC self-renewal was observed between BD I patients and healthy controls.

### *NLRP2* is the most significant DEG, at both the iPSC and NSC stages, in discriminating BD from healthy controls

**Figure 2:**
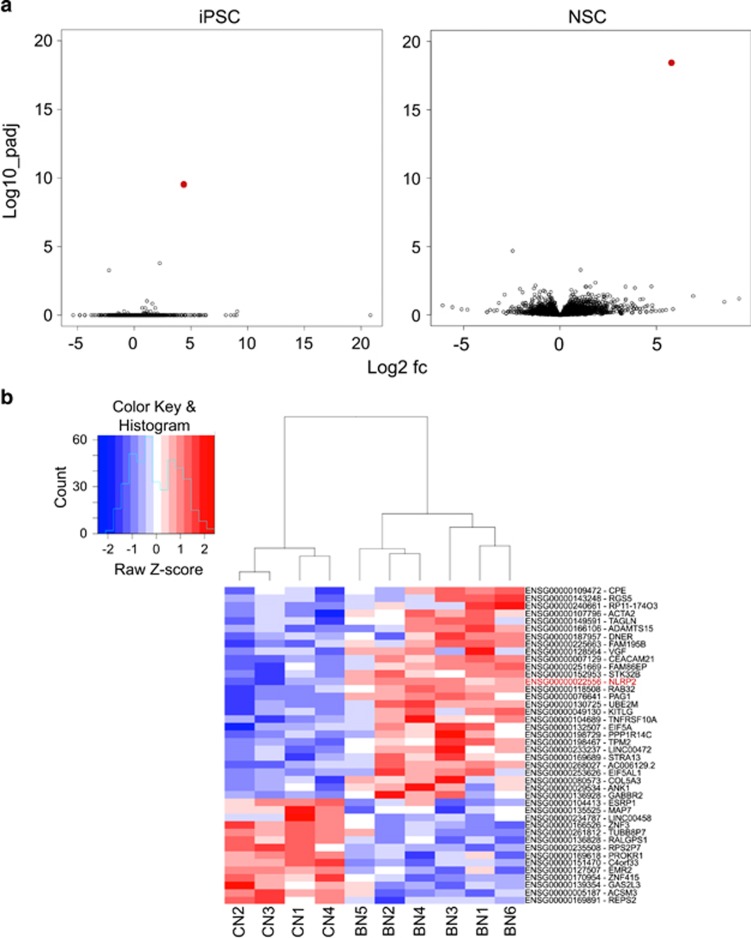
Differential gene expression signatures of BD-derived iPSC and NSC. (**a**) RNA-seq and DESeq analyses identified BD differentially expressed genes at both iPSC and NSC states. Volcano plots of log10 (adjusted *P*) versus the log2 (fold change between BD and controls) of all genes at respective stage. (**b**) Expression values of differentially expressed genes at NSC stage in 10 generated cell lines are shown in heat map format. The red, white and blue colours represent higher than average, close to average and lower than average expression of a particular gene, respectively, as measured by row standardised *Z*-scores. The rows are organised by hierarchical clustering using agglomerative clustering with complete linkage and Euclidian distance metric. BD, bipolar disorder; iPSC, induced pluripotent stem cell; NSC, neural stem cell.

To identify BD-associated genes, we examined the average differences in gene expression in samples from BD I patients relative to the healthy control iPSC and NSC cell lines, respectively. Examination of volcano plots revealed that only three genes exhibited a significant difference in expression between the six BD I and four control iPSC lines (adjusted *P*<0.05; Figure 2a, iPSC). In contrast to the lack of multiple DEGs in the iPSC state, there were 42 genes with significant expression differences between BD and controls in the NSC stage (adjusted *P*<0.05; Figure 2a, NSC). The heatmap and cluster analysis of NSC stage DEGs show the diversity in the gene expression profiles (Figure 2b). To extract the genes with the strongest difference in gene expression between BD and controls, the cut-off value for log2-fold change was set to 1.8. In total, 15 unique genes showed >1.8 log2-fold change between BD and control NSC (adjusted *P*<0.05), respectively (Tables 2A and B). Interestingly, one of these genes was differentially expressed at both states, that is, *NLRP2* (adjusted *P*=2.66 × 10^−10^).

**Figure 3:**
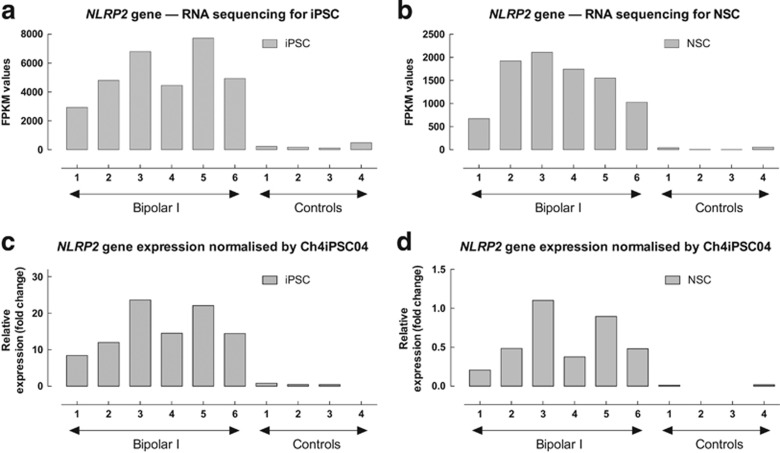
Illustration of altered *NLRP2* gene expression from six BD type I patients and four healthy controls. Relative changes in gene expression were calculated using the 2^−ΔΔCt^ method with *GUSB* and *RPLP* as reference genes. (**a**) RNA sequencing for iPSC. (**b**) RNA sequencing for NSC. (**c**) qPCR validation of the *NLRP2* gene in iPSC normalised by Ch4iPSC4. (**d**) qPCR validation of the *NLRP2* gene in NSC normalised by Ch4iPSC4. BD, bipolar disorder; iPSC, induced pluripotent stem cell; NSC, neural stem cell; qPCR, quantitative PCR.

A closer look at the RNA sequencing results for *NLRP2* gene expression revealed clear differences between BD I and control lines in both iPSC and NSC states (as illustrated in Figures 3a and b). Moreover, quantitative PCR analyses confirmed that expression of *NLRP2* is upregulated in both BD iPSC and NSC lines (Figures 3c and d). These results demonstrate that it is possible to identify several DEGs in the NSC stage by using an *in vitro* BD model system, and among these genes *NLRP2* was already affected at the iPSC state.

### Several cytoskeleton-associated genes are over-represented as DEGs of BD-NSC lines

In the 42 DEGs in NSC stage, cytoskeleton-associated genes are over-represented at the high levels of significance. For we believe the first time, we here report *TPM2* and *TAGLN* to be associated with BD. *TPM2* encodes for tropomyosin beta chain type 2, and is known to stabilise actin filaments and regulate calcium-dependent muscle contraction (Table 2A). *TAGLN* encodes for transgelin smooth muscle-alpha, a gene that regulates actin cross-linking and gelling protein, but the functional role of this protein is unclear. *ACTA2* (actin alpha-2 sm) has a role in cell motility, structure and integrity, being a major constituent of the contractile muscle. In addition, *MAP7* (microtubulin-7), *TUBB8P7* (tubulin beta 8 class VIII pseudogene 7), *ANK1* (ankyrin 1) and *COL5A3* (collagen type V alpha-3) were identified.

### Canonical pathway analyses of DEGs between control and BD NSCs identify abnormalities in TREM1, dopamine and GABA receptor signalling

We next addressed whether common networks or pathways were over-represented in the list of genes associated with the top DEGs. Ingenuity pathway analysis (Qiagen)—an application enabling the discovery, visualisation and exploration of molecular interaction networks in gene expression data—was performed (cut-off values: adjusted *P*<0.05, log2-fold enrichment 1.8) revealing enrichment for relevant gene networks: the canonical pathway important at both iPSC and NSC stages was revealed to be TREM1 (triggering receptor expressed on myeloid cells 1; *P*=0.00376 and *P*=0.0442, respectively; Table 3). The top scoring canonical pathway correlated with inflammation markers, for example, intrinsic prothrombin activation pathway (*P*=0.0179) followed by GABA receptor (P=0.0418), and dopamine receptor (*P*=0.0486) signalling. Furthermore, neurological diseases (*P*=0.00160 to 0.0129) and psychological disorders (*P*=0.00323 to 0.00129) were the top scoring diseases.

### *NLRP2* is not abnormally regulated in BD patient-derived adipocytes

We showed that also in the *in vitro* BD model, *NLRP2* transcripts are expressed higher in the BD-NSC-expressing cortical neuronal markers, when compared with those of healthy controls. We asked whether *NLRP2* transcript levels also expressed higher in the primary adipocyte cell cultures from five BD patients and 11 healthy control lines by quantitative PCR. It was found to be undetectable or very low levels of the transcript without any differences between the patients and controls (data not shown), indicating that the *NLRP2* transcript is not expressed in adipocytes. These results are in agreement with the GTEx portal (http://www.gtexportal.org), showing an absence of *NLRP2* gene expression from subcutaneous adipose tissue, indicating that the *NLRP2* gene is not expressed in adipocytes (see GTEx portal data set: www.gtexportal.org). These results demonstrate that in our *in vitro* BD model *NLRP2* is expressed in early uncommitted stem cells—equivalent to the cells of the inner cell mass of a blastocyst—and in cortically committed neural stem cells.

## Discussion

We established a human BD model system to understand when and which BD-associated genes manifest, by comparing RNA-expression levels at iPSC and NSC stages of BD I patients and healthy controls. The capacity of our *in vitro* iPSC-based BD model system regarding morphology, neuronal induction, NSC marker expression, and neuronal function are presented. Comparative RNA sequencing was applied and DEGs were identified at the iPSC and NSC stages.

Our study provides further evidence that BD represents a complex polygenetic developmental disorder. As the here-presented results in BD-specific altered RNA-expression levels and signal pathways indicate a high complexity, we will separately discuss each of our identified abnormalities with respect to clinical relevance for the aetiology, pathophysiology and diagnosis of BD. On the other hand, BD might be considered both a neurodegenerative and a neurodevelopmental disorder. Support for the former hypothesis is offered from reports of abnormal dendritic spine morphology and diminished hippocampal volume (as visualised by brain imaging in adult BD patients as well as in post-mortem tissue obtained from BD I patients), indicating neurodegenerative conditions.^42, 43^ However, owing to its well-documented heritability and alteration of dendritic spine morphology and immature axon growth, also seen in the autistic spectrum disorder,^44, 45^ as well as results from our current study, we suggest that bipolar patients have already a strong susceptibility toward the disorder in the early development of the nervous system *in utero*, or during the first few years of life. These considerations point at disease with very early onset, with its clinical phenotype becoming manifest much later.

### Early elevated expression of *NLRP2* and abnormal signalling pathway in immune responses: Does susceptibility for BD-associated inflammation start before birth?

Recently, the importance of inflammasomes in BD as well as in other central nervous system disorders (Alzheimer, stroke, brain and spinal cord injuries) have become recognised, indicated by high presence of inflammasomes in post-mortem brain samples.^46, 47^

NLRP2 belongs to the family of nucleotide-binding domain, leucine-rich repeat-containing proteins (NLRs, also known as NALP2, NBS1, PAN1 and PYPAF2), characterised by an N-terminal pyrin domain with innate immune functions, mediating activation of inflammasomes.^48, 49, 50, 51^ The NLRP2 inflammasome is a multiprotein complex, composed by NLRP2 and the adaptor protein apoptosis-associated speck-like protein, and it activates pro-inflammatory caspase-1, resulting in activation of IL-β.^51, 52^

In this pathological context, activation of inflammasomes has been described in microglia, macrophages and astrocytes,^47^ reviewed by Walsh *et al.*^53^ Interestingly, post-mortem frontal cortex obtained from BD patients shows a high amount of NLRP3 proteins (NLRP3 being a close relative to NLRP2), in comparison to bio-samples from healthy individuals.^46^ The presented highly significant difference in the NLRP2 transcriptome expression at the iPSC and NSC stages in our BD I patients cells indicates that inflammation-related process already starts at the very early embryonic development. In addition, the absence of high-level expression of NLRP2 transcripts in BD patient adipocytes indicates that the expression of NLRP2 differs among cell types. According to microarray data sets of NLRP2 expression, the human adult cortex (two donors at age 31) and the fetal telencephalon (two donors at ages 15 p.c.w. (post-conceptional weeks) and 16 p.c.w.) both express NLRP2 (Ref. http://human.brain-map.org/microarray/search/show?exact_match=true&seach_term=NLRP2&search_type=gene&donors=14380,15496,10021,9861,12876,15697, for fetal: 14751,12840). By the GTEx portal data set, NLRP2 was not detected in subcutaneous adipocyte but expressed in frontal cortex median RPKM: 0.731, sample #108: cortex RPKM: 0.635, sample #114: EBV transformed lymphocytes RPKM:2.970, sample #118. Thus, we need to search whether the described abnormal presence of proteins of the NLRP family in adult BD patients^46^ might have originated from an iPS cell stage, and whether abnormal regulation of *NLRP* genes during ontogeny induces a susceptibility that may become clinically relevant later during the patient’s lifespan.

As our finding of an early fetal expression of NLRP is completely new and unexpected, we can assume some prospective function of NLRP during human ontogeny. *NLRP2* has been shown to be expressed in oocytes and granulosa cells and identified as a maternal effect gene, and the depletion of *NLRP2* in zygotes led to early embryonic arrest.^54^ Our findings demonstrate markedly enhanced *NLRP2* transcript levels as early as at the iPSC stage of cells generated from BD I patients, and clearly distinguished a difference from healthy controls in NSC (Figure 2). However, this alteration did not affect either pluripotency of iPSC lines or neural induction. These results agree with previous findings indicating that overexpression of *NLRP2* in zygotes appears to lead to normal development before the blastocyst stage.^54^ However, further studies are required to describe the link of upregulated NLRP proteins during early human fetal neural development and adult brain neurones and glial cells.

Our study also revealed an enrichment of relevant gene networks in the TREM1 (Triggering Receptor Expressed on Myeloid cells 19) signalling pathway and showed that top scoring canonical pathway correlated with inflammation markers, for example, intrinsic prothrombin activation pathway, at both iPSC and NSC stages.

An increasing body of evidence points to the fact that inflammatory processes have critical roles in BD (see below). It is intriguing that a major modulator of inflammatory signalling as shown in this study is expressed at elevated levels in both iPSCs and NSCs generated from BD patients. Therefore, our results indicate that an abnormal regulation of inflammatory genes might start before birth, and thereby the susceptibility for BD-associated inflammation would more likely be directly genetically mediated, rather than environmentally induced.

### Do abnormal dopamine and GABA neurotransmitter systems in BD-NSC result in impaired neurotransmitter-regulated neural development or neuronal function?

The balance of dopamine, glutamate and GABA systems is an essential key for normal brain development and maintenance.^55^ Our pathway analysis shows enrichment for disease-associated genes to relate relevant networks and pathways. Although the top scoring canonical pathway correlated with inflammation markers, it was followed by GABA receptor and dopamine receptor signalling pathways. Persistent abnormalities in dopaminergic and GABAergic neurotransmitter systems in neuronal progeny might lead to aberrant functional brain oscillatory patterns within brain networks in patients with BD and major depressive disorder (MDD).^56^ Moreover, ontogeny of cells expressing dopamine and/or GABA receptors have not been clearly demonstrated in human NSC, but genes like *neurogenin2* and *Nurr1* are expressed in mouse NSC, which could start to differentiate into both pathways.^57, 58^

### Abnormal expression of cytoskeleton-associated genes: a prospective link between abnormal neural and cardiac development in BD and cardiovascular diseases?

We have also identified several cytoskeleton-associated genes as DEGs in the NSC stage. There is evidence that psychiatric disorders are connected with cytoskeletal genes by means of the transport of biological material, such as synaptic proteins along the axon.^59, 60^ The tubulin expression levels are often altered and dysfunctional in disease-specific regions such as the hippocampus and prefrontal cortex of schizophrenia and bipolar disorder patients.^61^ In our paper, *TPM2* is the strongest DEG followed by *TAGLN* among the cytoskeletal genes, and both can regulate *ACTA2*. ANK1 (protein encoded by the gene *ankyrin1*) links the integral membrane proteins to the spectrin–actin cytoskeleton and ion channels, being important for cell motility and synaptic activation. Hereditary human diseases due to defects or gene variation of ankyrins (1/2/3) are known to be linked with diseases such as cardiac arrhythmia and bipolar disorder.^30, 62^ Bipolar patients with psychotic episode are comorbid with cardiovascular diseases.^63^ Thus, the consequences of overexpressed *TPM2* and *TAGLN*, regulating actin filaments during the neural and cardiac development might give novel insights into the neural degeneration observed in bipolar disorder in post-mortem tissues,^42^ and might explain why BD is comorbid with cardiovascular diseases, and vice versa.

### Inflammation in mood disorders and future directions

The role of immunological factors in the pathophysiology and diagnosis of BD I has been discussed in relation to schizophrenia, and emphasis is put on an imbalance of inflammatory cytokines.^9^ Anti-inflammatory drugs exert proven clinical effects as shown in several randomised controlled trials in MDD.^64, 65, 66^ Treatments targeted against NLRP inflammasomes have been suggested to become useful against neuro-inflammation and in treatment of MDD.^47, 67^ Moreover, some forms of depression can be considered to represent microglial aberrations that should be treated with microglial stimulators or inhibitors.^68^ Also, if the inflammasome activation actually precedes psychopathology and is not essentially deactivated by psychopharmacological treatment, then inflammasome measures such as *NLRP2* may provide useful diagnostic biomarkers.

In conclusion, our discovery of an inflammasome activation in immature stem cells and neural cells at a very early stage of ontogeny indicates this feature of BD to have been set before environmental influences have had a reasonable chance to tune in. To this end, it might also be of importance to investigate the effects of different targeted treatments on the NLRP2 inflammasome. Studies are required to elucidate the role of NLRP2 in BD and its tentative use as a biomarker for this, and possibly other mood disorders.

### Limitation of the study

Sample size is small, although difference of NLRP2 expression is highly significant between BD I and control groups. BD I is a complex and polygenic disorder, which may make these findings difficult to generalise to all the BD I population. This is an *in vitro* modelling human system, not an *in vivo* study.

## Figures and Tables

**Table 1:** Fold changes in gene expression for some marker genes during neural induction shown for cell lines derived from BD patients and healthy controls

*Gene*	*DEG C_NSC_/C_iPSC_*		*DEG BD_NSC_/BD_iPSC_*	
	*Fold change (log2)*	P-value	*Fold change (log2)*	P*-value*
*Jarid1*	–2.15	3.60 × 10^–32^	–2.49	7.01 × 10^–65^
*Nanog*	–6.91	8.86 × 10^–9^	–6.18	1.99 × 10^–23^
*Tert*	–2.07	1.72 × 10^–18^	–1.80	1.77 × 10^–21^
*Foxg1*	7.92	7.68 × 10^–12^	8.19	6.82 × 10^–19^
*Lhx2*	9.64	4.00 × 10^–22^	8.64	1.83 × 10^–34^
*Otx1*	3.73	1.30 × 10^–6^	2.97	7.76 × 10^–7^
*Otx2*	1.84	5.54 × 10^–6^	2.00	4.60 × 10^–10^
*Pax3*	5.35	0.001	7.16	1.57 × 10^–8^
*Pax6*	6.40	6.80 × 10^–33^	5.95	2.57 × 10^–43^
*Pax7*	6.39	4.38 × 10^–5^	3.80	0.002
*Pou3f2*	5.26	3.10 × 10^–13^	4.81	9.08 × 10^–19^
*Sox1*	6.40	3.88 × 10^–9^	8.92	3.50 × 10^–16^
*Sox2*	1.02	0.0007	0.83	0.0006

Abbreviations: BD, bipolar disorder; DEG, differentially expressed gene; iPSC, induced pluripotent stem cell; NSC, neural stem cell.

**Table 2:** Differentially expressed genes between BD patients and healthy controls at (A) NSC and (B) iPSC stages, identified by RNA-seq

*Symbol*	*Name*	*Fold change (log2)*	*Adjusted* P*-value*
*A*			
* Upregulated DEGs at NSC stage*			
* NLRP2*	NLR family, pyrin domain containing 2	5.816	3.07 × 10^–19^
* TPM2*	Tropomyosin-2	1.067	0.51 × 10^–4^
* TAGLN*	Transgelin smooth muscle-alpha	1.829	0.00439
* CEACAM21*	Carcinoembryonic antigen-related cell adhesion molecule 21	4.740	0.00826
* ADAMS15S*	ADAM metallopeptidase with trombospondin type 1 motif, 15	3.397	0.00852
* ACTA2*	Actin alpha-2 sm	1.568	0.00852
* VGF*	VGF nerve growth factor inducible	2.480	0.0312
* DNER*	Delta/Noch-like EGF repeat containing	2.255	0.0316
* ANK1*	Ankyrin 1	1.658	0.0330
* COL5A3*	Collagen, type V, alpha-3	2.008	0.0387
* RGS5*	Regulator of G-protein signalling 5	2.594	0.0427
* PAG1*	Phosphoprotein membrane anchor with glycosphingolipid microd. 1	2.282	0.0427
* GABBR2*	Gamma-aminobutyric acid (GABA) B receptor 2	1.865	0.0474
* PPP1R14C*	Protein phosphatase 1, regulatory (inhibitor) subunit 14C	2.268	0.0485

*Downregulated DEGs at NSC stage*			
* RPS2P7*	Ribosomal protein S2 pseudogene 7	–2.455	2.06 × 10^–5^
* ADGRE2*	Adhesion G protein-coupled receptor E2	–2.075	0.0127
* MAP7*	Microtubulin-7	–1.038	0.0197
* LINC00458*	Long intergenic non-protein coding RNA 458	–1.941	0.0225
* TUBB8P7*	Tubulin, beta 8 class VIII pseudogene 7	–2.872	0.0330

*B*			
*Upregulated DEGs at iPSC stage*			
* NLRP2*	NLR family, pyrin domain containing 2	4.370	2.66 × 10^–10^
* EEF1A1P16*	Eukaryotic translation elongation factor alpha 1 pseudogene 16	2.250	1.61 × 10^–4^

*Downregulated DEG at iPSC stage*			
* RPS2P7*	Ribosomal protein S2 pseudogene 7	–5.380	5.30 × 10^–4^

Abbreviations: BD, bipolar disorder; DEG, differentially expressed gene; iPSC, induced pluripotent stem cell; NSC, neural stem cell.

**Table 3:** Top canonical pathways enriched in BD patients as recognised by ingenuity pathway analysis at iPSC and NSC stages, respectively

*Name*	P*-value*
*Top canonical pathways at iPSC stage*	
TREM1 signalling	0.00376
	
*Top canonical pathways at NSC stage*	
Intrinsic prothrombin activation pathway	0.0179
GABA receptor signalling	0.0418
TREM1 signalling	0.0442
Dopamine receptor signalling	0.0486

Abbreviations: BD, bipolar disorder; iPSC, induced pluripotent stem cell; NSC, neural stem cell; TREM1, triggering receptor expressed on myeloid cells 1.
