# Supplementary figures and images for: Early onset of inflammation during ontogeny of bipolar disorder: the NLRP2 inflammasome gene distinctly differentiates between patients and healthy controls in the transition between iPS cell and neural stem cell stages
Source: Transl Psychiatry. 2017 Jan 24;7(1):e1010–. doi: 10.1038/tp.2016.284 (PMC5545741; doi:10.1038/tp.2016.284)

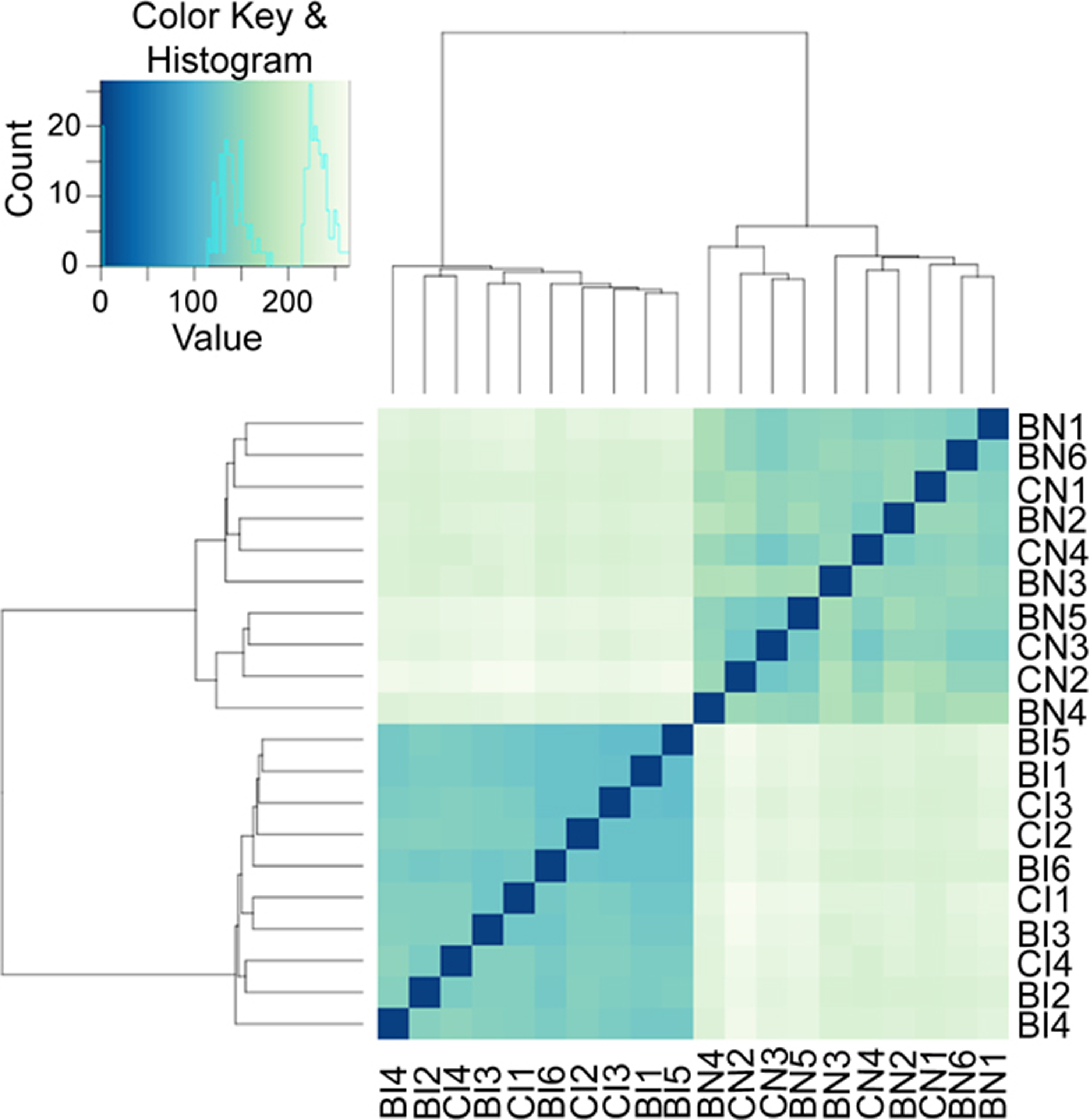

Supplement: Supplementary Figure 1 [file tp2016284x1.tif]
