# Supplementary material for: Early onset of inflammation during ontogeny of bipolar disorder: the NLRP2 inflammasome gene distinctly differentiates between patients and healthy controls in the transition between iPS cell and neural stem cell stages
Source: Transl Psychiatry. 2017 Jan 24;7(1):e1010–. doi: 10.1038/tp.2016.284 (PMC5545741; doi:10.1038/tp.2016.284)
Supplement: Supplementary Information [file tp2016284x2.docx]

**Supplementary Figure (in TIFF file)**

Expression values of transcriptomes at iPSC and NSC stage in 10 generated lines are shown in heatmap format. The dark blue, blue, and green/white colours represent higher than average, close to average, and lower than average similarity of a particular transcriptome, respectively, as measured by row standardized Z-scores. The rows are organised by hierarchical clustering using agglomerative clustering with complete linkage and Euclidean distance metric. All samples from individual stages, *i.e.* iPSC and NSC, have similar transcriptomes regardless of origin as revealed by clustering analysis.

**Supplementary Information 1**

All samples from individual stages, *i.e.* iPSC and NSC, have similar transcriptomes regardless of origin as revealed by clustering analysis.

**Supplementary Information 2 (in Excel data file)**

Differentially expressed genes between different stages in **(A)** healthy controls and **(B)** BD patients. A total of 3302 and 3187 transcripts, respectively, were differentially expressed at log2 fold enrichment 2 or higher in the control and BD cell lines between different stages.
